# Taking a Closer Look: “Evaluating Online Video Content for Rehabilitation after Distal Radius Fracture”

**DOI:** 10.3390/jcm13164691

**Published:** 2024-08-09

**Authors:** Roberta Laggner, Dominikus Huber, Timothy Hasenoehrl, Julia Sternik, Michaela Stoffer Marx, Rita Weber-Stallecker, Richard Crevenna, Gerhild Thalhammer, Stephan Heisinger

**Affiliations:** 1 Department of Orthopedics and Trauma Surgery, Medical University of Vienna, 1090 Vienna, Austria; roberta.laggner@meduniwien.ac.at (R.L.); gerhild.thalhammer@meduniwien.ac.at (G.T.); stephan.heisinger@meduniwien.ac.at (S.H.); 2 Department of Internal Medicine I, Division of Clinical Oncology, Medical University of Vienna, 1090 Vienna, Austria; 3 Department of Physical Medicine, Rehabilitation and Occupational Medicine, Medical University of Vienna, 1090 Vienna, Austriajulia.sternik@meduniwien.ac.at (J.S.); richard.crevenna@meduniwien.ac.at (R.C.); 4 Institute of Occupational Therapy, FH Campus Wien, 1100 Vienna, Austria; michaela.stoffer-marx@fh-campuswien.ac.at; 5 Institute of Therapeutic and Midwifery Science, Occupational Therapy, University of Applied Sciences Krems, 3500 Krems, Austria; rita.weber-stallecker@fh-krems.ac.at

**Keywords:** online content, distal radius fracture, rehabilitation, guidance, quality assessment

## Abstract

**Background**: Fractures of the distal radius are among the most common bone injuries, and their frequency is constantly increasing, leading to an elevated need for subsequent rehabilitation. This growing need has led to the emergence of online content aimed at providing guidance on rehabilitation. Nonetheless, unreviewed online content raises concerns about its reliability; therefore, the objective of this study was to evaluate the quality, reliability, and comprehensiveness of online videos concerning rehabilitation following a distal radius fracture. **Methods**: A total of 240 YouTube videos were screened, identifying 33 videos that met the inclusion criteria. These selected videos were evaluated by five independent experts from various professional groups, using the Global Quality Scale, the DISCERN reliability tool, and the JAMA Benchmark Score, as well as a structured set of questions to assess their comprehensiveness and coverage of pertinent aspects. **Results**: The observers’ assessment of the Global Quality Scale exhibited a broad spectrum of viewpoints, indicating considerable variability in evaluations. In most cases, therapy aligned well with the diagnosed condition, and most raters deemed the indication and instruction in the videos acceptable. A proportion of 87% of the videos was deemed suitable for home training by at least three raters. However, a concerning trend emerged, as potential risks and pitfalls were scarcely addressed. **Conclusions**: The moderate overall quality of the videos and the divergence in expert opinions highlight the need for a regulatory authority to ensure adherence to guidelines and maintain high-quality content. Additionally, our results raise concerns about the applicability of established assessment tools in this context.

## 1. Introduction

Fractures of the distal radius are among the most common fractures, representing around 18% of fractures in patients over 65 [1].

The incidence of fractures of the distal radius (DRF) has increased continuously across all age groups. Between 1999 and 2010, there was an annual increase of 2.0% in men and 3.4% in women [2,3,4,5]. The demographic displays a bimodal distribution characterized by peaks in incidence among postmenopausal women and elderly individuals due to factors such as osteoporosis and among young men due to sports injuries, accidents, or trauma [6].

To decide whether a fracture has to be treated conservatively or surgically, clinicians should consider various aspects, such as age, bone quality, comorbidities, functional demand, fracture characteristics, and associated injuries.

However for fractures that can be reduced by closed manipulation and then remain stable, conservative treatment with closed reduction and immobilization is appropriate [7].

It remains unclear which factors determine the outcome in terms of patient satisfaction. Some authors claim that patient satisfaction depends more on hand dominance and residual wrist pain than on range of motion [8]. Others have found grip power and return of wrist function to be the most significant factors [9,10]. Therefore, rehabilitation and physical therapy play crucial roles in restoring wrist mobility and strength post-treatment, aiming to facilitate a return to regular activities and minimize long-term complications, such as stiffness or chronic pain.

Physiotherapists and occupational therapists employ a tailored regimen of exercises, manual therapy techniques, and functional activities to improve joint mobility, muscle strength, and coordination. Additionally, they focus on reducing pain, swelling, and stiffness, while facilitating the individual’s return to daily activities and functional independence. [11,12]. Hence, early diagnosis, appropriate treatment, and comprehensive rehabilitation are pivotal in achieving optimal outcomes for individuals with distal radius fractures [13].

Starting with the COVID-19 pandemic-related restrictions in 2020, including lockdowns, the pre-existing shortage of traditional rehabilitation services was further aggravated. Consequently, efforts have been undertaken to address this shortfall by exploring alternative solutions, such as telerehabilitation as a substitute for in-person rehabilitation [14,15,16,17,18]. Although considerable efforts are being made to develop the above-mentioned substitute treatments, patients often have no or only limited access to proper rehabilitation due to the increasing incidence of injuries and the associated reduced capacity of rehabilitation centers [4,12].

The Internet has become one of the most significant sources of medical and health-related information for patients [19]. Nevertheless, 82% of patients have never or merely occasionally discussed the information they found online with their physician. YouTube is a non-peer-reviewed source of videos uploaded by a wide range of sources, and it is the world’s third most popular website, accounting for 60% of all video views online [20,21]. The platform is tremendously popular, with over 2 billion monthly active users. In actual terms, almost 43% of all global internet users access YouTube every month [22].

Examining existing non-professional content, such as user-generated videos, is important for several reasons. Firstly, this content is widely accessible to the general public and often serves as a primary source of information for many individuals seeking health advice. Understanding the nature, quality, and accuracy of this content is essential because it directly influences public health behaviors and perceptions. In contrast, summarizing virtual professional content found in the academic literature, while valuable, primarily benefits those within the professional or academic spheres. Professional content is usually peer-reviewed and adheres to high standards of scientific rigor, but it may not reflect the information that the general public is actually consuming and acting upon.

Therefore, by focusing on non-professional content, we can better understand the gaps, misinformation, and potential dangers present in the information landscape that is most accessible to and trusted by the average person. This approach allows for the development of targeted strategies to improve the quality of information available to the public, thereby enhancing overall public health literacy and outcomes [19,23,24].

Consequently, the aim of this study was to evaluate the scope, reliability, and quality of online videos on rehabilitation exercises after distal radius fractures using established assessment tools for online media, as well as to assess the suitability and reproducibility of these established assessment tools. We hypothesize that the currently available assessment tools are limited in providing an objective and reproducible assessment. Moreover, they are insufficient to ensure safe, high quality content as a monitoring tool.

## 2. Methods

The study structure was merely descriptive. Similarly to Heisinger et al., we used the search items “distal radius fracture physiotherapy”, “distal radius fracture rehabilitation”, “distal radius fracture ergotherapy”, and “distal radius fracture therapy exercises” on YouTube (www.youtube.com, accessed on 18 August 2021) to find physiotherapy exercise tutorials for patients with distal radius fracture [23,25]. The YouTube videos were sorted by the number of views, and the 60 most viewed videos for each search term were analyzed as described by Kocyigit et al. [26]. Consequently, 240 videos were evaluated, excluding off-topic videos, duplicates, videos in a language other than English, or otherwise unsuitable videos (e.g., poor video/audio quality). In order to meet these criteria, 33 were included in this study. The following information was recorded during screening: (1) the title of the video, (2) Universal Resource Locator, (3) number of total views, (4) number of likes, (5) number of dislikes and (6) sources of the videos. The video sources were classified as “non-medical”, “Health/Lifestyle Company”, “Physiotherapist”, and “Physician”. The sources were classified by the observers based on the information from the uploading party’s profile.

The videos included in this study were assessed by five independent observers. Raters were classified as ERGO1: occupational therapist 1; ERGO2: occupational therapist 2; PHYSIO: physiotherapist; SPOSCI: sports scientist; TRAUMA: trauma surgeon.

The observers evaluated the videos independently, and unlike other studies, no attempts were made to reach a consensus in case of discrepancies [26].

All observers were fluent in written and spoken English, and the medical staff were recognized experts in their respective fields. Three common rating and classification systems were used to evaluate the videos: the Global Quality Scale (GQS) was chosen to rate the quality of the included YouTube videos. This scale is a common tool for assessing the educational quality of health-related content online and ranges from 1 to 5 (1 = very poor quality; 5 = excellent quality) [23,26]. To rate the reliability of the included videos, the modified DISCERN instrument was used as a second scoring method, consisting of five binary yes/no questions, with each positive answer giving 1 point, thus allowing a maximum score of 5. Third, the videos were evaluated using the Journal of the American Medical Association (JAMA) benchmark criteria to determine accuracy and reliability. In addition, the observers rated the videos on a subjective basis (personal grading) using a scale of 1 to 5 (1 = excellent to 5 = inadequate). The videos were rated for sound and image quality on a scale of 1 to 5. In addition, observers noted whether advertising was present (yes/no) [19,26].

To assess the potential applicability of the selected videos, the observers had to answer eight questions (Table 1). The corresponding answers were recorded in binary form as yes (=1)/no (=0). The observers rated the videos independently of each other; no attempt was made to reach a consensus in the event of disagreement.

## 3. Statistical Analysis

All data were entered and processed for further analysis in MS Excel (Microsoft Corporation, 2018 Microsoft Excel, available at: https://office.microsoft.com/excel accessed on 18 March 2024), and statistical analysis was conducted via R Studio (RStudio “Prairie Trillium” Release (1db809b8, 16 May 2022) for macOS 3 2022.02.3 Build 492© 2024–2022 RStudio, PBC available under https://www.R-project.org/ accessed on 18 March 2024).

Descriptive analysis included the mean (for metric variables) and quantiles (ordinal variables). Hypothesis testing for differences in medians was conducted using Man–Whitney U tests, and associations were estimated via Spearman’s Rho. For rater agreement, Fleiss’ Kappa was calculated. Differences in distribution were tested via modified Chi-Square tests for multiple variables.

An alpha of 0.05 was assumed to constitute statistical significance. Where appropriate, confidence intervals are reported, using alpha = 0.05. In all cases, two-sided testing was performed.

## 4. Results

**Figure 1:**
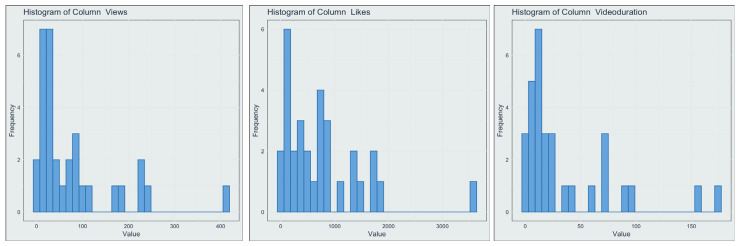
Histograms for views, likes, and duration of the examined videos show severe skewness and outliers at the top ends of the data, highlighting the heterogeneous nature of YouTube material.

Five healthcare professionals rated 33 videos on YouTube. At the time of rating, the videos had between 1449 and 499,247 views and between 37 and 5825 likes (Table 2). Duration varied from just above 1 min to over 20 min. Confidence intervals for the medians could not be calculated for views and duration due to severe skewness of the distribution (Figure 1). For likes, it is also likely not a good estimate, since all three variables show obvious outliers at the top ends. There was no significant correlation between video duration, likes (*p* = 0.346), and views (0.867).

**Figure 2:**
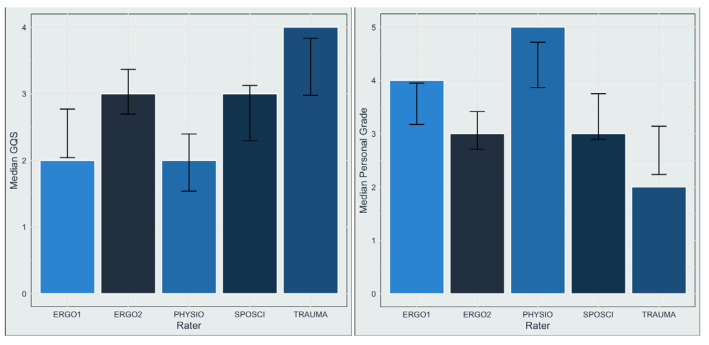
Each rater’s median GQS (**left panel**) and personal grade (**right panel**). In both cases, the trauma surgeons assigned better scores to the videos than occupational therapist 2 and the sports scientist (ERGO1: occupational therapist 1; ERGO2: occupational therapist 2; PHYSIO: physiotherapist; SPOSCI: sports scientist; TRAUMA: trauma surgeon).

The median rater assessment for all videos was “moderate quality (GQS = 3). Across all raters, “poor quality” (GQS = 1) was rated in 17%, “generally poor” (GQS = 2) in 28%, “moderate quality” (GQS = 3) in 28.5%, “good quality” (GQS = 4) in 19.4%, and “excellent quality” (GQS = 5) in 7.3% of the cases. The personal grades (1 = best grade, 5 = worst grade) assigned by each rater to each video mirror these results (Figure 2): in total, 9.1% of videos were graded with 1, 18.8% with 2, 26.1% with 3, 21.8% with 4, and 23% with 5.

The Kruskal–Wallis test for median GQS and personal grading suggests a significant difference in median scores among raters (for both variables, the Bonferroni adjusted *p* < 0.001). Graphically, the median GQS scores of the trauma surgeon, as well as the personal gradings for the videos, were better, while occupational therapist 2 (ERGO2) and the sports scientist (SPOSCI) rated the videos worse.

Fleiss’ Kappa for five raters for GQS is 0.04 (*p* < 0.001), and for personal grading, it is −0.02 (*p* = 0.015), indicating that there is no rater agreement on overall video quality.

After a Bonferroni correction, there was no significant correlation between GQS or personal grade and the length of the video.

The GQS correlated significantly and strongly with the answer to the question “Does therapy match diagnosis?” for the sports scientist (r = −0.34, *p* = 0.007) and the occupational therapist 2 (r = −0.40; *p* = 0.035). The other raters showed non-significant correlations, with the same direction ranging from −0.21 to −0.50, indicating that answering the question with no (=0) is associated with a lower overall GQS.

**Figure 3:**
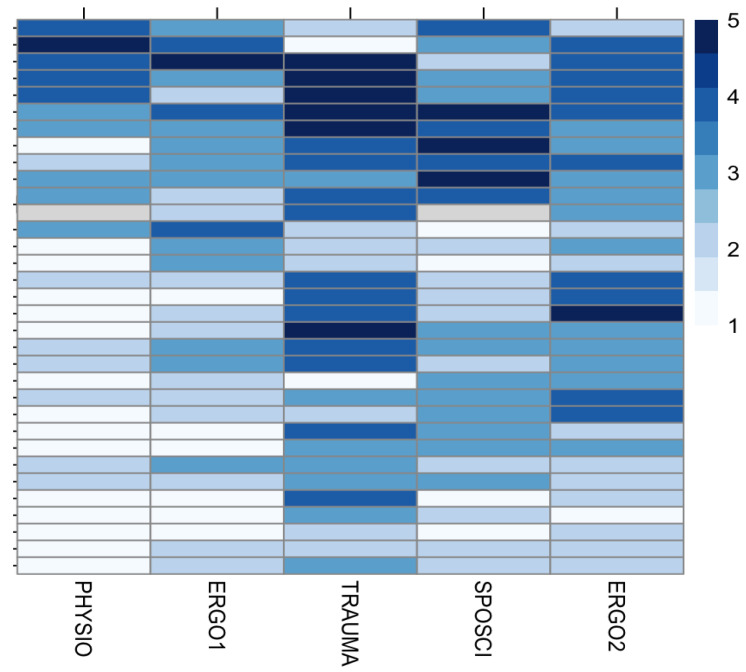
GQS scores for each video (rows) from all raters (columns) were ordered by the logarithm of the respective views of each video (*y*-axis).

The GQS scores for each video from all raters, ordered by the logarithm of the respective views of each video, are shown in Figure 3. The trauma surgeon (column 3) rated the videos significantly better (darker blue) than the physiotherapist and occupational therapist 1 (columns 1 and 2). While graphically a higher GQS is associated with more views (darker colors on one end of the y-axis, lighter colors on the other), there was no significant correlation detectable between rating and views, likes, or video duration.

**Figure 4:**
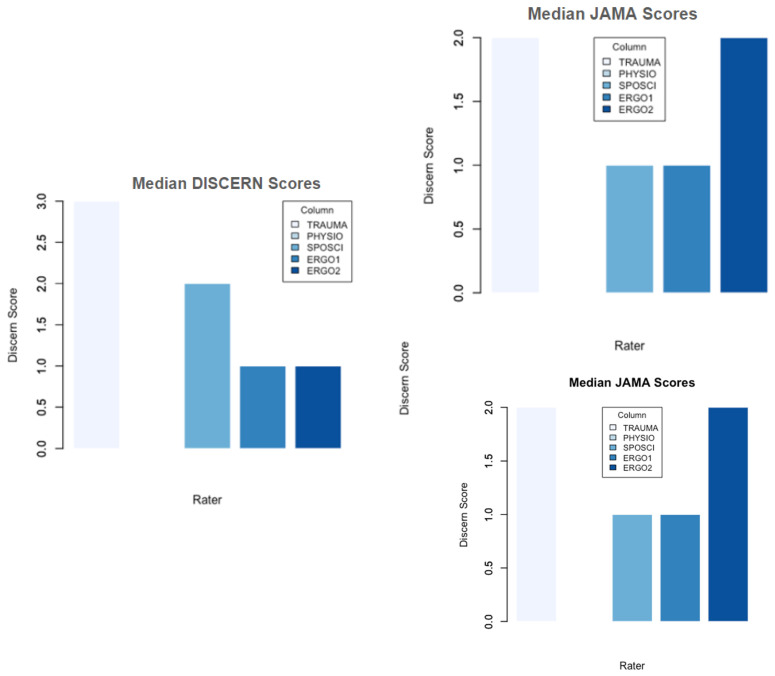
Median scores for JAMA (**right panel**) and DISCERN tool (**left panel**) by rater (from left to right in each panel: TRAUMA = trauma surgeon, PHYSIO = physiotherapist, SPOSCI = sports scientist, ERGO1 = occupational therapist 1, ERGO2 = occupational therapist 2). “PHYSIO” gave a rating of 0, hence no bar is displayed.

The median DISCERN score for all raters over all videos was 1, ranging from 0 (minimum) to 5 (maximum). Overall, 38 videos (23%) scored 0 points in the DISCERN tool from all authors, 54 (32.7%) scored 1 point, 47 (28.5%) scored 2 points, 21 (12.7%) scored 3 points, and only 3 videos (1.8%) scored 4, and 2 (1.2%) scored 5 points. The trauma surgeon gave a median score of 3 and awarded the score five once, whereas the other raters awarded a score of 1 or 2 (Figure 4). The physiotherapist showed the greatest variance in scoring, with a median of 0, but also one score of 4 and one of 5. The occupational therapists both awarded a median score of 1, while the sports scientist’s median DISCERN score was 2 (Figure 3). Three videos stood out with a median DISCERN score of 3 across raters. Overall rater agreement for the DISCERN tool was very low (Fleiss’ Kappa = −0.0296, *p* = 0.342). A slight, albeit not statistically significant, agreement could be found between the sports scientist and the occupational therapist 1 (Fleiss’ Kappa 0.10, *p*-value = 0.387).

For the JAMA scoring, the median was 1.5 for all raters and videos. In total, 36 (21.8%) videos were given 0 points, 76 (46.1%) were given 1 point, 50 (30.3%) were given 2 points, 3 (1.8%) were given 3 points, and none were given the full 4 points in the JAMA score. Again, three videos were rated better than the rest across raters, with a median JAMA score of 2 (videos 2, 18, and 51). Notably, these videos were rated better with the DISCERN tool. Again, rater agreement overall was poor, with Fleiss’ Kappa = −0.066 (*p*-value = 0.0849).

However, individual rater pairs showed more consistent answers: the trauma surgeon and occupational therapist 2 showed substantial agreement, with a Fleiss’ Kappa of 0.649 (Bonferroni-corrected *p*-value < 0.001), and the physiotherapist and occupational therapist 2 had a Fleiss’ Kappa of 0.600 (Bonferroni-corrected *p*-value < 0.001).

## 5. Sound and Video Quality

**Figure 5:**
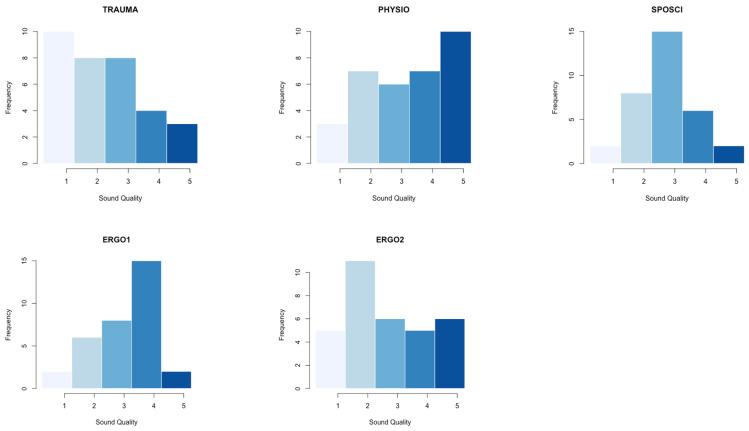
Absolute quantities (*y*-axis) of sound quality grading (*x*-axis) for each grader (TRAUMA = trauma surgeon, PHYSIO = physiotherapist, SPOSCI = sports scientist, ERGO1 = occupational therapist 1, ERGO2 = occupational therapist 2). Grades are sorted from left to right from 1 (light blue) to 5 (dark blue). The darker the color the higher the grade.

Overall rater agreement was slight but statistically significant for both sound (Fleiss’ Kappa = 0.111; *p* < 0.001) and video (Fleiss’ Kappa = 0.133; *p* < 0.001) quality. The median grade for both dimensions was 3 (average quality). The physiotherapist assigned significantly worse grades to the sound and video quality than the trauma surgeon (Kruskal–Wallis test for sound quality *p* = 0.006, for video quality *p* < 0.001). At the same time, the other graders showed a more balanced assignment graphically (exemplary of the sound quality assessment in Figure 5). As presented earlier, the sound and video quality showed no significant association with JAMA, DISCERN, or GQS.

Absolute quantities of sound quality are shown in Figure 5. The physiotherapist assigned significantly worse grades to the sound and video quality than the trauma surgeon (Kruskal–Wallis test for sound quality, *p* = 0.006; for video quality, *p* < 0.001). In contrast, the other graders showed a more balanced assignment graphically.

**Figure 6:**
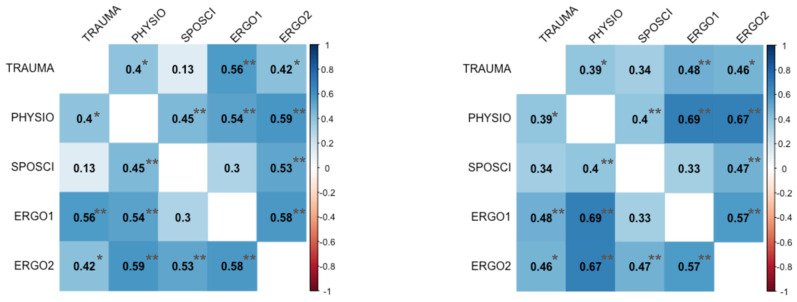
Spearman’s Rho correlation matrix for grading of video quality (**left panel**) and sound quality (**right panel**) for each rater (TRAUMA = trauma surgeon, PHYSIO = physiotherapist, SPOSCI = sports scientist, ERGO1 = occupational therapist 1, ERGO2 = occupational therapist 2) and with indicators for *p*-values < 0.05 (*) and *p* < 0.01(**). The correlation coefficients for both dimensions are strictly greater than zero (blue background), moderate (light blue) to large (dark blue), and statistically significant for all effect sizes greater or equal to 0.40.

**Figure 7:**
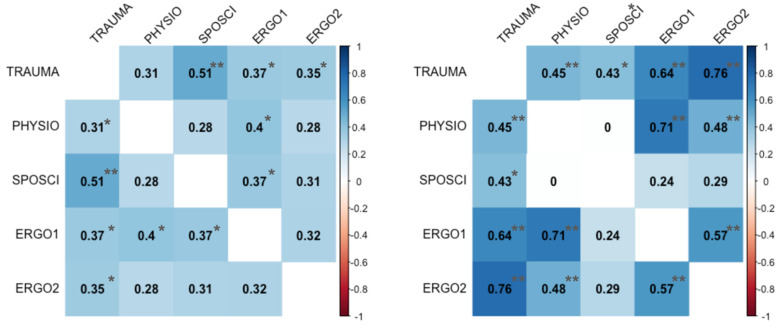
Spearman’s Rho correlation matrix for DISCERN (**left panel**) and JAMA (**right panel**) scores for each rater (TRAUMA = trauma surgeon, PHYSIO = physiotherapist, SPOSCI = sports scientist, ERGO1 = occupational therapist 1, ERGO2 = occupational therapist 2) and with indicators for *p*-values < 0.05 (*) and *p* < 0.01(**). The correlation coefficients for both dimensions are strictly greater than zero (blue background) and range from moderate (light blue) to very large (dark blue).

In contrast to visual examination, Kruskal–Wallis’ test, and Fleiss’ Kappa statistic for rater agreement, Spearman’s Rho correlation coefficient was positive for all rater pairings regarding sound and video quality—many of them statistically significant (Figure 6). The same is true for the JAMA and DISCERN scores (Figure 7). In summary, Fleiss’ Kappa showed poor rater agreement, while Pearson’s correlation coefficient displayed a strong, positive, and statistically significant association with the rater’s judgment.

**Figure 8:**
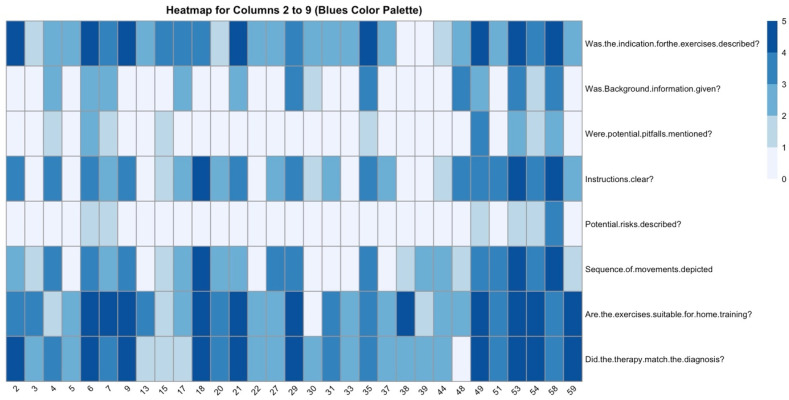
For each video (columns, video-ID on the *x*-axis), the absolute number of raters answering a question (rows) with yes is color-coded as dark blue = “all raters answered yes” and white = “no rater answered yes”.

The raters answered a structured set of questions regarding the information contained in the video. The indication for exercises was described in most videos according to the majority of raters (Figure 8): for 26 videos (83.9%), three or more raters answered this question with yes. Similarly, in 67.6% of the videos, at least raters agreed on a yes for the question “instructions clear?”. For the questions “potential pitfalls mentioned?” and “potential risks described?” this percentage was only 12.9% and 3.2%, respectively, indicating that only very few videos were assessed to answer these questions. For the questions “Background information provided?” and “Sequence of movements depicted,” the percentage of videos in which three or more raters would answer these questions with “yes” was 34.5% and 45.2%, respectively.

Regarding whether the exercises can be carried out at home, at least three or more raters deemed 87% of videos suitable for home training. Fleiss’ Kappa was poor as a measure of rater agreement on these questions (0.06, *p*-value = 0.220). A Pearson’s Rho correlation matrix showed weak to moderate, but not statistically significant (Bonferroni-corrected), correlation among raters on all questions.

For the final question, “Did the therapy match the diagnosis?” a slight, statistically significant rater agreement could be detected, with Fleiss’ Kappa = 0.13 (*p*-value = 0.029). The trauma surgeon answered “yes” for 31 videos (93.9%) and the physiotherapist did so for only for 11 videos (33.3%). The sports scientist (yes in n = 26 videos, 78.8%), occupational therapist 1 (yes in n = 22 videos, 66.7%), and occupational therapist 2 (yes in n = 29 videos, 87.9%) showed more similar results. On average, including the physiotherapist, in 72.1% of videos, the therapy was believed to match the diagnosis.

## 6. Discussion

In the present day, patients no longer rely solely on their physicians for information on their diseases and, in particular, the invasive procedures proposed to treat these diseases. They are turning to YouTube to gather additional information, aiming to take a more proactive stance in their healthcare choices. However, given that YouTube lacks a peer-review system, physicians and patients should remain mindful of the varying quality of information and the diverse sources providing health-related content. Although easily and quickly available, the reliability and accuracy of online sources remain controversial and raise significant concerns, especially for patients with a lack of medical training [27,28,29].

To our knowledge, this is the first study to investigate the quality of online videos regarding exercise therapy after distal radius fracture. Several studies on the quality assessment of health issue-related topics have already been carried out in the past, with the authors coming to similar conclusions, namely that the quality of online videos varies greatly and is not particularly high [23,24,26,30].

On a positive note, therapy aligned well with the diagnosed condition in most cases, showcasing a congruence between treatment and identified issues. While indications and instructions in most videos were deemed satisfactory by a consensus of observers, a significant oversight emerged concerning the scarce mention of potential risks and pitfalls. This specific issue has already been discussed in previous publications [23,24,25,31].

It is somewhat troubling that the popularity metrics, such as likes and views, failed to align with professional video quality assessments, elucidating the disparity between public perception and expert evaluation in this domain. There were significant differences in overall assessment among the different disciplines, with a clear tendency for physiotherapists to be more severe in their assessment compared to trauma surgeons. However, despite this contrast, there was a strong positive correlation, suggesting a common basis for assessing these disciplines. Interestingly, we observed that there was no rater agreement in regard to GQS and personal grading, which is contrary to our previous findings, especially in regard to GQS [25]. This finding may, however, be attributed to the variety of the observers’ professions and their respective varying approaches to this topic, which consequently highlights that there seems to be rather little consensus on the ideal rehabilitation exercises after distal radius fractures among the different professions. Altogether, this is a relevant finding, since it emphasizes the need to establish and validate consensus rehabilitation guidelines that are supported by all professions in the best interest for the patient.

The overall low rater agreement in regard to the applied scores (e.g., GQS, JAMA, DISCERN) possibly suggests that the existing assessment tools are insufficient to evaluate videos in an objective and reproducible manner. Nevertheless, it has to be clearly stated that this finding may be due to the observers’ different professions.

Most strikingly, however, they often overlooked crucial safety-related aspects. We observed that while the majority of videos neglect pointing out potential risks or pitfalls of the displayed exercises, their overall quality rating, e.g., GQS, ranges from poor to excellent. This underscores the danger of relying solely on a cumulative score, which may mask crucial deficits, such as potentially risky aspects, amidst an otherwise favorable rating.

## 7. Conclusions

Overall, the available YouTube videos focusing on rehabilitation exercises after distal radius fractures were generally of moderate quality, as determined by their Global Quality Scale (GQS) scores. Surprisingly, this grading and overall subjective evaluations showed no correlation with likes or views, making it challenging for non-medical individuals to identify reliable content. Our results show that inter-rater agreement is generally low using the established scores on this subset of videos, which raises concerns in regard to their applicability and reproducibility. Furthermore, these findings highlight the demand for consensus guidelines on rehabilitation exercises after distal radius fractures. Additionally, most videos failed to address potential risks and complications, which we consider pivotal. However, such shortcomings are not reflected by cumulative scores, which in turn raises questions about their suitability to evaluate online video content on rehabilitation exercises. Future research should focus on developing and evaluating standardized, evidence-based virtual rehabilitation programs. This includes creating professional, peer-reviewed video content and exploring interactive elements such as virtual reality and telehealth. Additionally, comparing patient outcomes between professional and non-professional content can help identify the most effective rehabilitation methods.

In conclusion, there is a pressing need for peer-reviewed, high-quality videos that encompass crucial aspects of rehabilitation while remaining easily understandable for the average patient. Furthermore, a more reproducible and objective scoring system to assess online rehabilitation exercise videos is necessary to ensure consistency and reliability.

## Figures and Tables

**Table 1:** Questions asked to assess applicability of the selected videos.

	Yes (1)	No (0)
Are indications for the exercises described?		
Is there background information?		
Are potential pitfalls mentioned?		
Are the instructions clear?		
Potential risks described?		
Is the sequence of movements depicted adequately?		
Is it possible at home?		
Does therapy match the diagnosis?		

**Table 2:** Descriptive statistics of all videos’ views, likes, and video duration, with percentiles.

	Views	Likes	Duration Sec
Minimum	1449	37	65
25% Percentile	20,788	237.25	299
Median	37,158	630	408
75% Percentile	95,851	1140.5	517
Maximum	499,247	5825	1239
Lower limit 95% confidence interval of the median	n/a	494.9	n/a
Upper limit 95% confidence interval of the median	n/a	1048.2	n/a

## Data Availability

Data are available from the authors on reasonable request.
